# Oral Hygiene Practices and Awareness of Pregnant Women about the Effects of Periodontal Disease on Pregnancy Outcomes

**DOI:** 10.1155/2022/5195278

**Published:** 2022-06-06

**Authors:** Noora Mohammed Eissa Bushehab, Jayadevan Sreedharan, Sesha Reddy, Jovita D'souza, Hossam Abdelmagyd

**Affiliations:** Gulf Medical University, Ajman, UAE

## Abstract

**Objectives:**

Adverse pregnancy outcomes, such as preterm low birth weight (PTLBW), is a severe public health issue that needs to be dealt with by educating the general female population, dental practitioners, and gynecologist. One of the major contributing factors is periodontal disease, which accounts for about 18.2% of all PTLBW cases worldwide, and prevention of the same could reduce the occurrence of PTLBW babies. This study aimed to assess the oral hygiene practices and awareness of pregnant women about the effect of periodontal disease on pregnancy outcomes.

**Methods:**

A descriptive cross-sectional study was conducted where 400 pregnant women after consent were requested to complete an anonymous, self-administered, structured questionnaire with twenty-one close-ended questions.

**Results:**

Only 5% of participants in this study reported that gum diseases during pregnancy lead to preterm labor or low birth weight babies. About 70.7% of pregnant women mentioned that they did not use any interdental cleaning aids, and 54.5% did not use mouthwashes.

**Conclusion:**

Tooth brushing practices among pregnant women were good; however, there was an overall lack in the use of adjunctive aids. The study highlighted a deficiency in the awareness of pregnant women regarding the association between periodontal disease and adverse pregnancy outcomes.

## 1. Introduction

Periodontal disease is a chronic inflammatory process of microbial origin that affects periodontal tissues. It provokes the destruction of supporting tissues of the teeth, such as the gingiva, periodontal ligament, and alveolar bone [1–4]. It starts with gingival inflammation known as gingivitis, and it appears clinically as swelling due to the increase in gingival crevicular fluid production rate, redness, accompanied by bleeding from the gingival sulcus on gentle probing. When the inflamed gingiva is neglected and left untreated, the inflammation progresses and extends to the underlying periodontal tissues causing loss of the tooth's connective tissue fiber attachment, loss of alveolar bone, apical migration of junctional epithelium along the root surfaces, mobility of the teeth, and eventually teeth loss. This severe form of inflammation is called periodontitis [1–4]. Periodontal disease is prevalent in both developed and undeveloped countries, and it affects around 90% of the worldwide population [2, 3]. Dental plaque is the main etiological factor for periodontal disease. However, there are many risk factors associated with periodontal disease like improper or inadequate oral hygiene, smoking, hormonal disturbance, and lack of education [1–4].

The link between systemic diseases and periodontal diseases is well known. Periodontal bacteria, which is a constant potential source of infection, when it enters the bloodstream, causes activation of the immune cells. These activated cells produce cytokines (inflammatory biological signals) that have a highly adverse effect throughout the body system and have been linked to cardiovascular diseases, cerebrovascular diseases, peripheral arterial diseases, diabetes mellitus, and adverse pregnancy outcomes [2, 4–6].

During pregnancy, gingivitis and periodontitis are the most common oral diseases observed due to the high level of estrogen and progesterone hormones [7–9]. There is an increase in periodontal pathogens like *Prevotella* species since these bacteria require the mentioned steroidal hormones for their growth. These bacteria affect the tissues around the teeth by increasing the inflammatory level and hence the bleeding tendency of the tissue. Therefore, pregnant women are vulnerable to gingival inflammation even with relatively low levels of plaque [7–9].

There is an increasing body of literature, which have examined the association, between periodontal diseases and adverse pregnancy outcomes. It is evident that periodontal disease is an important risk factor for several common adverse pregnancy outcomes [7–9]. The translocation of periodontal pathogens to the fetoplacental unit or the effect of inflammatory mediators, like interleukin-1 (IL-1), IL-6, IL-8, tumor necrosis factor-*α* (TNF-*α*), or prostaglandin E_2_ (PGE_2_), explains the possible link of periodontal diseases and various adverse pregnancy outcomes [7–9]. As a result, pregnant women are more susceptible to challenging maternal and prenatal outcomes, such as preeclampsia, preterm labor, foetal growth restriction, and low birth weight babies [7–11]. The link between maternal periodontitis and adverse pregnancy results is essential because preterm labor and low birth weight are major causes of infant death [10, 11].

WHO (World Health Organization) states that, around fifteen million babies are born annually before completion of gestation (gestation less than thirty-seven weeks), and the number keeps elevating. Approximately 5–8% of births in 184 countries are preterm. The rate of increase was 9.8% and 10.6% in the years 2000 and 2014, respectively. The main affected areas were Asia and Sub-Saharan Africa. Multiple factors, including demographic and socioeconomic status, have been directly linked to preterm birth, such as the age of the pregnant lady, increasing number of pregnancies, history of preterm birth, gestational hypertension, antepartum bleeding, and urinary tract infection [12].

In addition to this study, the second major problem is LBW babies, which is defined by WHO as weight below 2500 g. This is a result of preterm birth, intrauterine growth restriction (IUGR), or both preterm birth and IUGR and is also exponentially increasing. LBW babies are vulnerable to many diseases, including growth retardation, infectious/bacterial diseases, and delay in development in childhood, and eventually, in later stages of life [12].

Therefore, this study aimed to assess the oral hygiene practices and awareness of pregnant women regarding periodontal disease and its effect on pregnancy outcomes in Ajman, UAE. The objectives of the study were to determine the oral hygiene practices among pregnant women including tooth brushing habits, use of adjunctive aids, and readiness to seek periodontal treatment during and after pregnancy. Secondly, to assess the knowledge and awareness of pregnant women regarding the correlation of periodontal disease and pregnancy outcomes, specifically preterm low birth weight (PTLBW) babies. And finally, to correlate the frequency of visits for dental checkups, oral hygiene practices, and the knowledge and awareness of pregnant women regarding the link between periodontal disease and pregnancy outcomes.

## 2. Methods

A cross-sectional study was conducted among pregnant Emirati and non-Emirati women. The sample size for this study was 400. This study was approved by the IRB of Gulf Medical University and was conducted at the Gynecology Department of Thumbay University Hospital in Ajman. Data were collected using a questionnaire. The draft questionnaire was developed by reviewing the available literature regarding the knowledge and practices about periodontal disease and pregnancy outcomes. The content validation of the questionnaire was carried out before finalizing the questionnaire. The questionnaire had five domains, with 21 closed-ended questions regarding sociodemographic data, pregnancy information, awareness about periodontal disease and its effect on pregnancy outcomes, and oral hygiene practices. Data were collected in patient waiting areas of the Gynecology Department of Thumbay University Hospital from December 2020 to February 2021. Pregnant women in all the trimesters of pregnancy, who were willing to participate and sign the consent form, were included in the study. The filled up questionnaire was collected and checked for the completeness of information. Data were entered into an Excel datasheet and transferred to IBM SPSS 27 for statistical analysis. Descriptive and analytical statistics were used to analyze the data. Frequency and percentage of all variables were taken. The association was assessed using the chi-square test. Fisher's exact test was used wherever the chi-square test was not applicable.

## 3. Results

This research was conducted to assess the oral hygiene practices and awareness of pregnant women about the effect of periodontal disease on pregnancy outcomes. A total of 400 pregnant women participated in this study. Most of the participants, 136 (34.0%), were in the age group between 30 and 34 years. The mean age of participants with a standard deviation was 31.3 ± 5.6 years. Nearly 238 (59.5%) participants were from Eastern Mediterranean Region (EMR) and 162 (40.5%) from other regions. Of the total participants, 291 (72.8%) had higher education. Regarding the occupation of the participants, 242 (60.5%) were homemakers followed by 143 (35.8%) employed, and 15 (3.8%) were students.

Of the total 400 participants, 101 (25.3%) reported that they suffered from bleeding, while tooth brushing. Regarding the history of recession in the gum, 53 (13.3%) said that they did experience recession the gum during pregnancy. No family history of periodontal disease was reported by the majority 325 (81.7%) participants.

**Figure 1:**
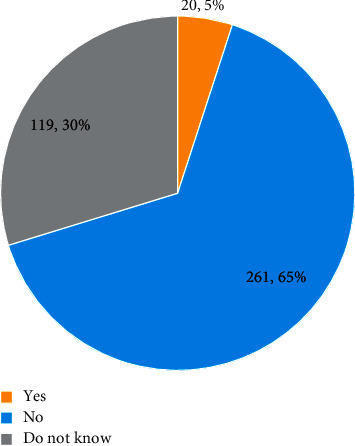
Awareness regarding gum disease as a risk factor for preterm labor.

**Figure 2:**
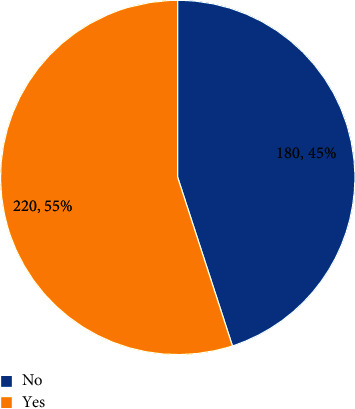
Awareness about the relationship between periodontal disease and pregnancy outcomes.

In the present study, 395 (98.8%) knew how to brush their teeth, 168 (42%) reported that oral hygiene should be increased during pregnancy. The effect of gum disease on the outcome of pregnancy was asked to the participants. Only 20 (5%) respondents said that gum diseases during pregnancy lead to preterm labor or low birth weight (Figure 1). Regarding the relationship between periodontal disease and pregnancy, 180 (45%) participants said that there are no association between periodontal disease and pregnancy (Figure 2).

 119 (29.8%) women said that their last visit to the dentist was three months within pregnancy. 391 (97.8%) reported that they brush their teeth daily, 117 (29.3%) said that they practice interdental cleaning, and 182 (45.5%) reported that they use mouth wash. Among these participants, 265 (66.4%) reported that they would seek periodontal treatment if they were ever diagnosed with periodontal disease during pregnancy. Among these participants, 383 (96.2%) said they would seek periodontal treatment if they were ever diagnosed with the periodontal disease after delivery (Table 1).

The association between their practice of oral hygiene and opinion of increasing oral hygiene during pregnancy was asked to the participants. All variables except undergoing treatment after delivery were significantly associated with their opinion. The opinion is associated with their practice. The details are given in Table 2.

Participants were asked regarding their oral hygiene practice, and if they had premature labor and low birth weight babies in the past. Six items included under the practice of oral hygiene were brushing teeth daily, frequency of brushing, using interdental cleaning, using mouth wash, undergoing treatment during pregnancy, and undergoing treatment after delivery. None of the practice variables showed statistical significance except undergoing treatment during pregnancy (*P* < 0.05). 64% of women mentioned that they would undergo treatment during pregnancy, whereas 33.5% mentioned that they would not undergo treatment during pregnancy. About 8.7% of women had preterm low birthweight babies in the past (Table 3).

## 4. Discussion

The mother's profile, habits, general and oral health awareness, and behaviours can have an impact on the health of babies. In order to reduce treatment procedures, current medical and dental practices emphasize on health promotion and prevention rather than the treatment [13, 14]. This strategy has variety of benefits for the community's health and economy.

In our study, we found that although tooth brushing practices were good, there was an overall lack in the use of interdental cleaning aids and mouthwash. This demonstrates an inadequacy of awareness among pregnant women regarding the recommended oral hygiene practices, which involves lack of understanding of the importance of interdental hygiene and use of mouthwash in general and also during pregnancy. A higher percentage of women who frequently visited the dentist used interdental cleaning aids, where 37.8% of women who visited the dentist within 3 months used inter-dental aids, while only 17.7% of women who visited the dentist more than 1 year ago used interdental aids. Similarly, among those who visited dental checkup regularly 53.8% used mouthwash, 47% who visited the dentist after 6–9 months used mouthwash, while only 39.2% of women used mouthwash whose last dental checkup was more than 1 year ago. This lack of knowledge and, hence, practice could be due to irregular visits for dental care during pregnancy, which is similar to the study conducted by Ramamurthy et al. [15].

Several studies have shown that pregnant women do not frequently visit the clinic for a dental checkup [16, 17]. In our study, only 29.8% of pregnant women visited the dental office for checkup regularly. This group had good practices of brushing frequency in which 93.3% brushed their teeth more than once a day. In women who visited the clinic for a dental checkup more than 1 year ago, only 77.9% of them brushed their teeth more than once a day. Frequent visits to the dentist facilitate better oral hygiene practices due to constant education, motivation, and monitoring, which in turn reflects in behaviour of patients, hence showing better oral hygiene outcomes [18]. Various studies have shown a positive correlation between oral health knowledge and practices [19, 20]. In our study, we found a lack of knowledge in many aspects. It was also noticed that the awareness of individuals increased with their regular visits for dental checkup. Our findings indicate that there is a need to provide oral hygiene education and instructions to an expectant woman, especially in the group that does not have regular dental checkups.

When estimating the readiness of women to undergo treatment during pregnancy, we found that only 66.4% of women mentioned that they would undergo treatment during pregnancy, while this percentage rose sharply when considering treatment after delivery, where 96.2% would undergo treatment after pregnancy.

Although 55% of participants in this study were aware about the relation of pregnancy and gum disease, 65.3% of participants in this study said that gum disease does not lead to preterm labor. This points to the fact that there is a limited understanding regarding the relationship of pregnancy and gum disease, and there is a need for imparting this knowledge not only to pregnant women but also to the gynecologists, since they regularly follow-up with pregnant women and can, hence, incorporate the practice of regular dental visits before, during, and after pregnancy for the overall health and maintenance of health of the mother and child leading to a healthier community at large.

Oral hygiene practices can be influenced by awareness. Pregnant women in this study who were willing to undergo dental treatment, if diagnosed with periodontal disease during pregnancy, were the ones who demonstrated good knowledge regarding periodontal diseases. This can be seen in the group who were aware regarding the association of gum disease and pregnancy (78.2%) more than who did not know (53.9%). This is similar to the study conducted by Hashim [21]. However, women who experienced preterm labor in the past were less willing to undergo treatment during pregnancy than those who did not experience preterm labor (51.4% and 67.9%, respectively). This could be due to the fear factor and cultural beliefs of undergoing something that might have an effect on the foetus due to their previous unpleasant experience and definitely lack of knowledge and awareness [20, 22].

Only 5% of the participants in this study reported that gum diseases during pregnancy lead to preterm labor or low birth weight babies, and the majority said that gum disease does not affect the pregnancy outcomes. This result highlighted the presence of a serious gap, which has to be addressed by organizing awareness programs for women and all related health care providers.

Unfortunately, majority of oral disease preventive initiatives are aimed at those who have already developed the condition, a practice known as late prevention, with little emphasis on early prevention. Pregnant women should be at the center of these programs for early prevention, as pregnancy is the ideal time for parents to study and practice healthcare, not only for the treatment of problems that arise but also to learn about their child's dental health [23, 24]. This concept must be incorporated into the family health strategy, ensuring dental treatment for all family members, especially for women who are planning pregnancy, during pregnancy, and after delivery. The dental health care practitioners and gynecologists must be made aware of the relationship of periodontal health and pregnancy and, therefore, be able to provide treatment for the overall benefit for the women and their pregnancy outcomes, which will be reflected positively on the community health and the economy at large. Preterm births represent one of the greatest health challenges of the 21st century. Health policymakers in many countries have not prioritized preterm birth as a health problem due to the lack of data showing the toll of prematurity and associated disabilities. Measures should be taken to increase the awareness about this association, thereby attempting to prevent the occurrence of adverse pregnancy outcomes.

This study was conducted during the COVID-19 pandemic, which could have impacted the data regarding the patient's visit to the dentist. The percentage of patients visiting the dental office for routine checkups and follow-ups dropped drastically during the pandemic.

## 5. Conclusion

The participants exhibited good tooth brushing practices, but there was a lack in the use of interdental cleaning aids and mouthwashes. The study results highlighted an overall deficiency in the awareness of pregnant women regarding periodontal disease and its relation to adverse pregnancy outcomes, which in turn impacted their oral hygiene practices. Women who regularly visited the dentist for a dental checkup showed a comparatively higher awareness and slightly better practices.

### 5.1. Work Goals

  Organize awareness programs for women and all related health care providers such as medical practitioners, gynecologists, nurses, and dentists regarding the correlation of periodontal disease and pregnancy outcomes.
  Educate gynecologists and medical practitioners regarding the importance of frequent dental checkups for women during pregnancy and encouraging an interdisciplinary health care approach.
  The concept of “early prevention” of periodontal disease must be incorporated into family health care strategies, ensuring dental treatment for all family members, especially for women who are planning pregnancy, during pregnancy, and after delivery.
  Develop a strategy of teaching and implementing good oral hygiene practices like proper tooth brushing techniques and emphasizing on interdental cleaning, which should be an integral part of daily oral hygiene in all individuals in the community, especially during pregnancy.

## Figures and Tables

**Table 1:** Association between oral hygiene practice and last dental checkup (*N* = 400).

Oral Hygiene Practice	Group	Last dental checkup						Total	*P*
		Within 3 months		6 to 9 months ago		>1 year			
		No.	%	No.	%	No.	%		
Brush teeth daily	No	—	—	2	2.0	7	3.9	9	^*∗∗*^
	Yes	119	100.0	98	98.0	174	96.1	391	
Frequency of brushing	One time	8	6.7	9	9.0	40	22.1	57	<0.001
	>1 time	111	93.3	91	91.0	141	77.9	343	
Use interdental cleaning	No	74	62.2	59	59.6	149	82.3	282	<0.001
	Yes	45	37.8	40	40.4	32	17.7	117	
Use mouth wash	No	55	46.2	53	53.0	110	60.8	218	<0.05
	Yes	64	53.8	47	47.0	71	39.2	182	
Brush teeth after every meal	No	83	69.7	71	71.0	148	81.8	302	<0.05
	Yes	36	30.3	29	29.0	33	18.2	98	
Material used to clean teeth	Brush, paste	62	52.1	54	54.0	122	67.4	238	^*∗∗*^
	Brush, paste, floss	10	8.4	7	7.0	6	3.3	23	
	Brush, paste, floss, mouthwash	47	39.5	38	38.0	48	26.5	133	
	Others	—	—	1	1.0	5	2.8	6	
Undergo treatment during pregnancy	No	16	13.6	36	36.0	82	45.3	134	<0.001
	Yes	102	86.4	64	64.0	99	54.7	265	
Undergo treatment after delivery	No	2	1.7	3	3.0	10	5.6	15	^*∗∗*^
	Yes	116	98.3	97	97.0	170	94.4	383	

**Table 2:** Association between oral hygiene practice and knowledge of oral hygiene practices during pregnancy (*N* = 400).

Oral hygiene practice	Group	Oral hygiene should be increased during pregnancy				Total	*P*
		Yes		No			
		No.	%	No.	%		
Frequency of brushing	One time	15	8.9	29	16.2	57	<0.05
	>1 time	153	91.1	150	83.8	343	
Use interdental cleaning	No	96	57.1	150	84.3	282	<0.001
	Yes	72	42.9	28	15.7	117	
Use mouth wash	No	75	44.6	115	64.2	218	<0.001
	Yes	93	55.4	64	35.8	182	
Undergo treatment during pregnancy	No	35	20.8	77	43.0	134	<0.001
	Yes	133	79.2	102	57.0	265	
Undergo treatment after delivery	No	5	3.0	8	4.5	15	NS
	Yes	161	97.0	171	95.5	383	

**Table 3:** Association between oral hygiene practice and history of premature labor and low birth weight babies in the past (*N* = 400).

Oral hygiene practice	Group	Ever had premature labor and low birth weight babies in the past				Total	*P*
		No		Yes			
		No.	%	No.	%		
Brush teeth daily	No	7	77.7	2	22.3	9	NS^*∗*^
	Yes	357	91.3	34	8.7	391	
Frequency of brushing	One time	52	91.2	5	8.8	57	NS
	>1 time	312	91.0	31	9.0	343	
Use interdental cleaning	No	258	91.5	24	8.5	282	NS
	Yes	105	89.7	12	10.3	117	
Use mouth wash	No	197	90.4	21	9.6	218	NS
	Yes	167	91.8	15	8.2	182	
Undergo treatment during pregnancy	No	117	87.3	17	12.7	134	<0.05
	Yes	247	93.2	18	6.8	265	
Undergo treatment after delivery	No	13	86.7	2	13.3	15	NS
	Yes	349	91.1	34	8.9	383	

## Data Availability

The data used to support the findings of this study are available from the corresponding author upon request.
